# Successful treatment of metastatic adrenocortical carcinoma in the spine: A case report and literature review

**DOI:** 10.1097/MD.0000000000018259

**Published:** 2019-12-10

**Authors:** Shuzhong Liu, Xi Zhou, An Song, Zhen Huo, Yipeng Wang, Yong Liu

**Affiliations:** a Department of Orthopaedic Surgery, Peking Union Medical College Hospital, Peking Union Medical College and Chinese Academy of Medical Sciences; b Department of Endocrinology, Key Laboratory of Endocrinology, National Health and Family Planning Commission; c Department of Pathology, Peking Union Medical College Hospital, Chinese Academy of Medical Science & Peking Union Medical College, Beijing, China.

**Keywords:** cement augmentation, metastatic adrenocortical carcinoma, radiation therapy, radiofrequency ablation, spine, surgical treatment, targeted PD-1 therapy

## Abstract

**Rationale:**

Adrenocortical carcinoma is a rare aggressive type of cancer whose prognosis is poor, particularly for metastatic entities. Metastatic adrenocortical carcinoma in the spine is a rare disease with no standard curative managements yet. The objective of this study is to report a very rare case of spinal metastases of adrenocortical carcinoma successfully managed by combination of cement augmentation, radiotherapy together with adjuvant programmed cell death 1 (PD-1) therapy. The management of these unique cases has yet to be well-documented.

**Patient concerns:**

A 42-year-old woman presented with a 3-month history of continuous and progressive back pain. The patient, who had been diagnosed of right pheochromocytoma, received surgical treatment of right adrenalectomy 14 months ago in another hospital, followed by no further treatment.

**Diagnosis:**

Magnetic resonance imaging of spine showed vertebral pathological fracture of L1, spinal cord compression secondary to the epidural component of the L1 mass, with increased metastatic marrow infiltration of the right L1 vertebral body, which presented as a solid tumor. Postoperative pathology confirmed the diagnosis of spinal metastases of adrenocortical carcinoma.

**Interventions:**

The patient underwent cement augmentation via a posterior approach, radiotherapy, radiofrequency ablation of psoas major muscle occupying lesions, right chest wall, liver and kidney recess together with adjuvant PD-1 therapy.

**Outcomes:**

The patient's neurological deficits improved significantly after the surgery, and the postoperative period was uneventful at the 6-month and 1-year follow-up visit. There were no complications associated with the operation during the follow-up period.

**Lessons:**

Combined efforts of specialists from orthopedics, urology, interventional radiology, radiotherapy, pathology, endocrinology, and medical oncology led to the successful diagnosis and management of this patient. Metastatic adrenocortical carcinoma of the spine, although rare, should be part of the differential diagnosis when the patient has a history of adrenal carcinoma and presents with back pain, myelopathy, or radiculopathy. We recommend the posterior approach for total excision of the spinal metastatic adrenocortical carcinoma when the tumor has caused neurological deficits. Osteoplasty by cement augmentation, radiotherapy, and targeted PD-1 therapy may also be good choices for treatment.

## 1 Introduction

Spinal metastasis of adrenal cortical carcinoma (ACC) is exceedingly rare with only few cases having been reported previously; thus, there is still short of imaging proof.^[1–4]^ In general, ACC has poor prognosis and high recurrence rates, even with aggressive resection.^[5,6]^ To date, management of malignant adrenocortical carcinoma of spine has remained under evaluation, with no standard criteria. Herein, we are presenting an analysis of a rare case of spinal metastases of adrenocortical carcinoma with paravertebral extension treated with cement augmentation, radiotherapy, radiofrequency ablation of psoas major muscle occupying lesions, right chest wall, liver and kidney recess together with targeted programmed cell death 1 (PD-1) therapy. In the short term, the patient's conditions improved significantly postoperatively. After reviewing pertinent literature, we discussed common diagnosis and management considerations in patients with metastatic spinal adrenocortical carcinomas.

## 2 Case report

In December of 2016, a 40-year-old woman with no notable medical history presented to another hospital, with incidental elevation of blood pressure. Abdominal computed tomograhy (CT) revealed a right adrenal mass (6.3 cm × 6.2 cm × 6.0 cm). An excision of the adrenal mass was performed, and pathology revealed features consistent with pheochromocytoma. Subsequently, she was discharged from the hospital with a prescription for 10 mg of phenoxybenzamine twice a day to control her blood pressure.

The patient presented to our institution in February 2018. Upon examining and questioning the patient, she stated she has been experiencing progressive back pain for approximately 3 months, after a sudden collision. The pain in her back can reach 8 points using the visual analogue scale and cannot be alleviated with rest and hot compresses. Initially, the patient attributed the pain to her overwork and thus did not seek medical attention. The patient denied experiencing any loss of consciousness, chest palpitations, diaphoresis, paresthesia, headaches, fatigue, and facial flushing. She also denied convulsions, nausea, vomiting, and other constitutional symptoms. Upon further questioning, she recalled she had been diagnosed of right pheochromocytoma, received surgical treatment of right adrenalectomy in another hospital, followed by no further treatment. No pertinent family history was identified, including, hypertension, cancer, and congenital birth difficulties.

**Figure 1:**
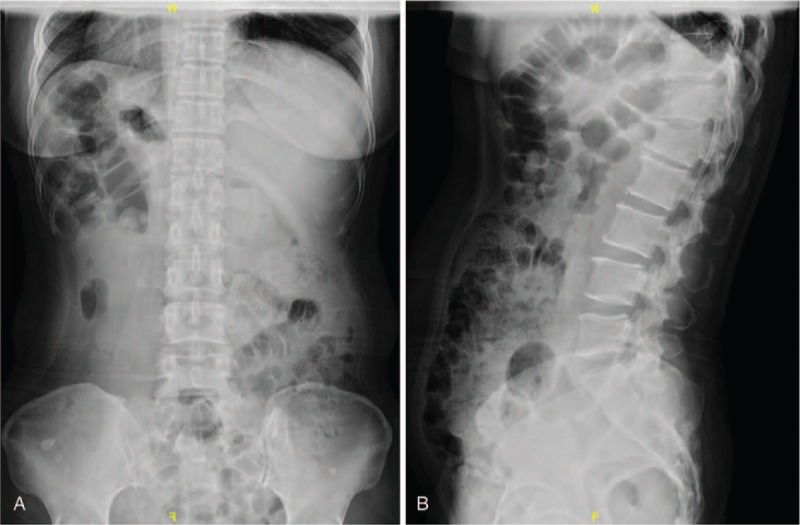
(A and B) Preoperative x-rays revealing vertebral fracture of L1 with high suspicion of metastatic spinal tumors.

**Figure 2:**
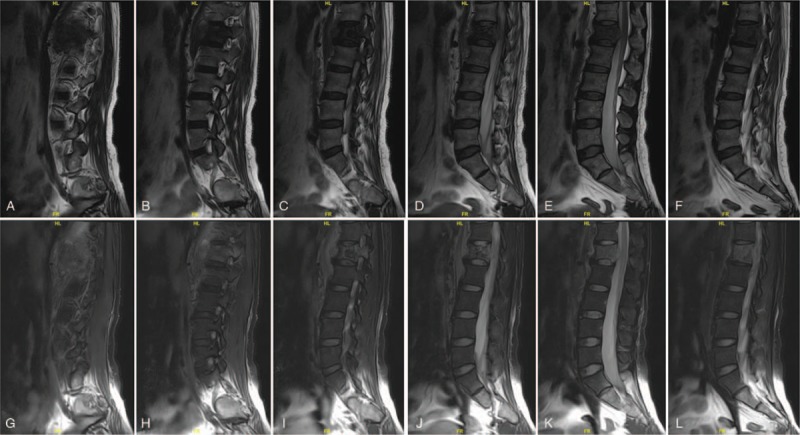
(A–L) Preoperative sagittal magnetic resonance imaging scan revealing the density of soft tissue, obvious bony destruction in the L1, and spinal cord compression caused by metastatic adrenocortical carcinoma, with increased metastatic marrow infiltration of the vertebrae and paravertebral regions.

**Figure 3:**
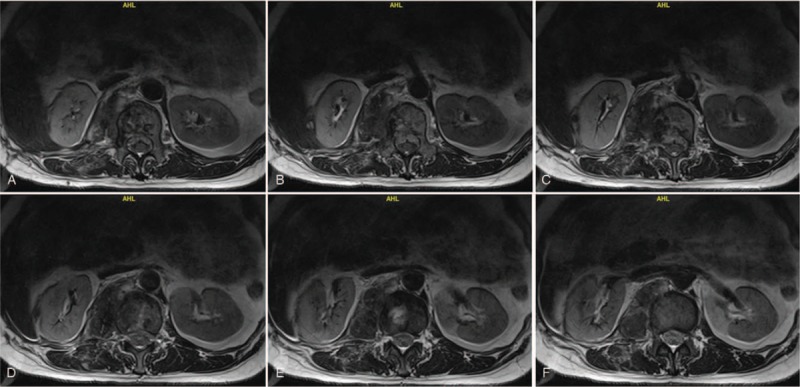
(A–F) Preoperative transverse magnetic resonance imaging images showing the metastatic adrenocortical carcinoma in L1 and spinal cord compression caused by metastatic adrenocortical carcinoma.

**Figure 4:**
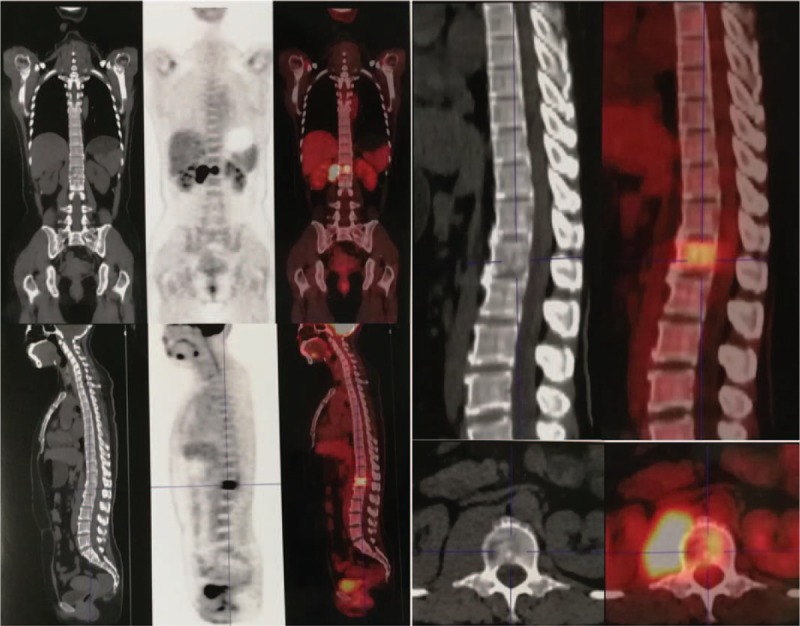
Positron emission tomography-computed tomography revealed malignant tumors of the L1 with paravertebral involvement.

Upon physical examination, the patient showed decreased sensation to pin-prick and fine-touch sensation of right lower extremity and exhibited an 5-/5 strength in the right lower limb. Deep tendon reflexes revealed normal for both knee jerk and Achilles tendon reflexes bilaterally. Ataxia was absent. Cranial nerves, mini mental, and the rest of the neurological examination showed no abnormalities. Routine laboratory tests were ordered, including electrolytes, liver and kidney function tests, and complete blood count. Genetic evaluations for RET, VHL, SDHB, SDHC, SDHD, an 24-hour urine fractionated catecholamines were ordered. Abdominal CT, spinal magnetic resonance imaging (MRI), and fluorodeoxyglucose-positron emission tomography/CT (FDG-PET/CT) were ordered to visualize the metastatic lesions, assess the stability of the vertebral column, and to aid in the formulation of a surgical approach. Preoperative hemodynamic and cardiovascular assessments included electrocardiogram, echocardiogram, and chest x-ray. The results of the laboratory studies were almost within normal range, except that the 24-hour urine fractionated catecholamine revealed a elevated level of 10.56 μg/24 h (normal: 1.74–6.42 μg/24 h), and adrenocorticotropic hormone elevated to 109 pg/mL (normal: <46 pg/mL). Genetic investigation was negative for RET, VHL, SDHB, SDHC, SDHD mutations. x-ray showed vertebral pathological fracture of L1 (Fig. 1A and B). MRI of the lumbar spine revealed widespread abnormal signals of L1 vertebrae in keeping with diffuse metastatic infiltration (Fig. 2A-L). MRI of spine showed progressive spinal cord compression secondary to the epidural component of the L1 mass, with increased metastatic marrow infiltration of the L1 vertebral body, which mimicked a solid tumor (Fig. 3A-F). The tumor infiltrated through the L1 vertebral body into the right pedicle and posterior elements. Extraosseous spread into the right lateral aspect of the epidural space extending posteriorly, resulting in compression of the nerve root. The spinal cord is displaced and spinal cord stenosis is significant at L1 level (Fig. 3A-F). A PET/CT scan revealed multiple osteolytic lesions of the spine, especially prominent in L1 and paravertebral region. PET/CT also demonstrated multiple suspicious metastases in the L1, paravertebal region, psoas major muscle occupying lesions, right chest wall, right liver and kidney recess (Fig. 4).

**Figure 5:**
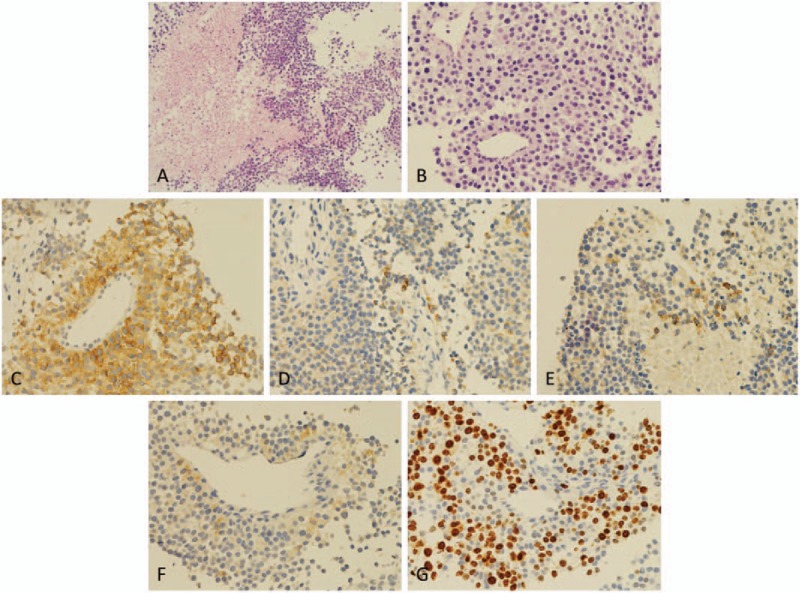
(A and B) Posteroanterior (PA) and lateral x-ray image of the lumbar spine obtained postoperatively.

First, we performed CT-guided puncture and biopsy of right psoas major muscle mass. After consultation with pathology department, the pathology report of the right psoas major muscle mass and the primary adrenal tumor both confirmed adrenocortical carcinomas, not consistent with pheochromocytoma (Fig. 5A-G). After full preoperative evaluation and multidisciplinary discussion, cement augmentation was performed to destroy the functional tumor and stabilize the spine under local anesthesia.

**Figure 6:**
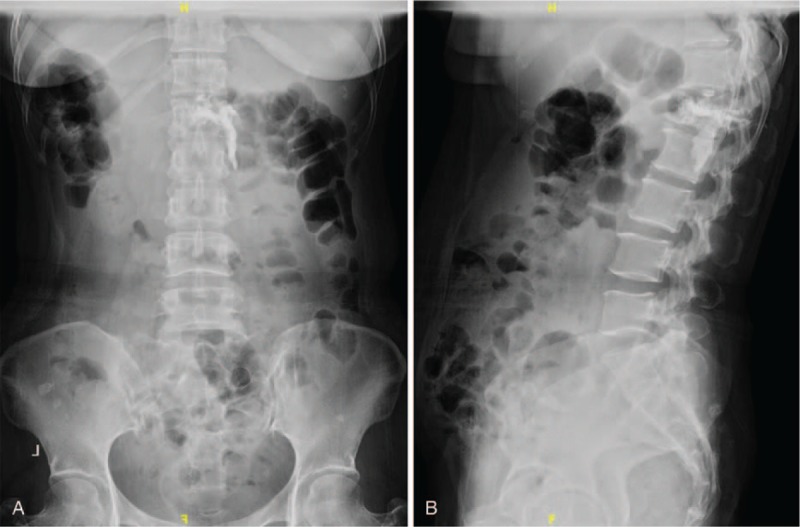
Pathologic histology of paraspinal tumors in the right psoas major. (A and B) Microphotography showing characteristic nests of tumor cells separated by vascular septa (Zellballen) with cells showing significant nuclear pleomorphism with prominent nucleoli (H&E, original magnification 100× and 200×). (C) Synaptophysin immunostaining shows strong, diffuse cytoplasmic staining in the tumor cells. (D) Melan-A immunostaining shows focally positive staining in the tumor cells. (E and F) EMA and α-inhibin immunostaining shows negative staining in the tumor cells. (G) Ki-67 immunostaining shows 30% Ki-67 positive cells. Ki-67 staining is localized in the tumor nuclei.

**Figure 7:**
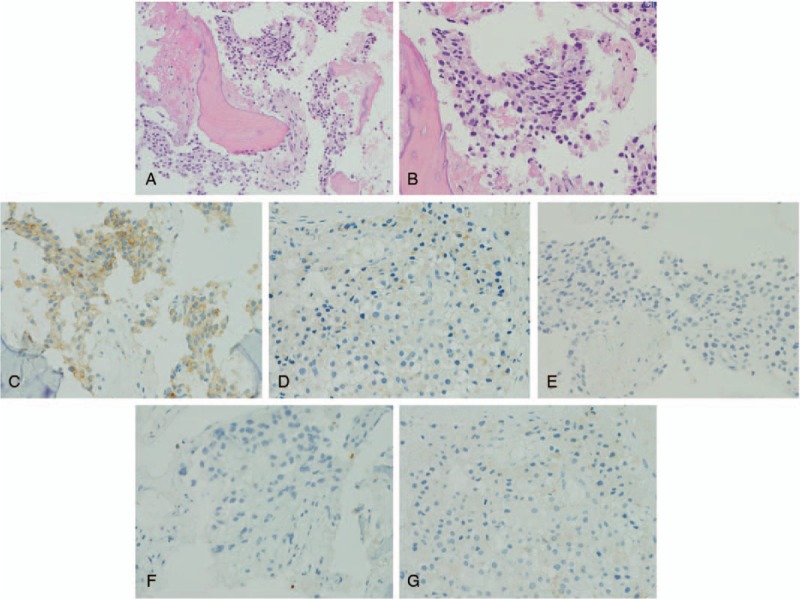
Pathologic histology of spinal metastatic adrenocortical carcinoma. (A and B) Microphotography showing characteristic nests of tumor cells separated by vascular septa (Zellballen) with cells showing significant nuclear pleomorphism with prominent nucleoli (H&E, original magnification 100× and 200×). (C) Synaptophysin immunostaining shows strong, diffuse cytoplasmic staining in the tumor cells. (D) Melan-A immunostaining shows focally positive staining in the tumor cells. (E–G) Chromogranin A, S100, and α-inhibin immunostaining shows negative staining in the tumor cells.

In brief, percutaneous vertebroplasty at L1 was performed according to the original surgical plan. For the posterior approach, we used C-arm for perspective positioning, L1 vertebral lesion was identified as surgical target, and the right L1 pedicle puncture point was located. Then 2% lidocaine was used for local infiltration anesthesia, and the puncture needle was inserted through the cannula. Under the C-arm fluoroscopy, the L1 vertebral lesion was penetrated through the right pedicle of the L1, and then we finished biopsy of the L1 mass. Then we send out the tissue for pathological examination. Bone cement for vertebroplasty was introduced. Under the perspective, the 8.0-mL cement of L1 was slowly pushed through the putter, and the biopsy passage was closed. Fluoroscopy confirmed the good dispersion of bone cement. The operation was successful and intraoperative bleeding was about 30 mL. The blood pressure and heart rate were stable during the operation. Postoperatively, x-ray of the spine revealed part of bone cement leaked into the paraspinal region (Fig. 6A and B). Postoperatively, the patient experienced significant pain relief. Pathological examination confirmed the diagnosis of malignant adrenocortical carcinoma (Fig. 7A-G).

One week after the operation, the patient's right lower extremity muscle strength improved to grade V compared to the preoperative status, grade V-. Moreover, VAS score of her back pain improved to 0–1 points compared to the preoperative status, 8 points. As the patient did not develop severe adverse effects, she was discharged and was monitored as an outpatient. Following wound healing, the patient underwent rehabilitation therapy. Radiotherapy, radiofrequency ablation, and targeted PD-1 therapy were also conducted. Radiofrequency ablation of psoas major muscle occupying lesions, right chest wall, liver, and kidney recess was completed within 1 month after the operation. Radiotherapy of the right psoas major muscle (60Gy/15f, 5f/w) and spine (45Gy/15f, 5f/w) was performed in the second month after the operation, respectively. Six months after the operation, she received the first cycle of targeted PD-1 therapy. The postoperative 6-month and 1-year follow-up visit showed no tumor progression and no new symptoms.

## 3 Discussion

Adrenocortical carcinoma is a rare malignancy with poor prognosis. The incidence of ACCs is approximately 0.7 to 2.0 cases per 1,000,000 individuals per year, and the metastatic forms account for a small fraction of all cases.^[1,2]^ In literature, ACCs are more commonly diagnosed during the fourth and the fifth decades of life for the sporadic form.^[3,4]^ Metastatic lesions can be localized or systemic, with the latter being marginally more common.^[5–11]^ Localized metastases tend to involve surrounding structures (including pancreas, spleen, liver, intestine, and retroperitoneum), whereas systemic forms occur most frequently in the lungs and liver, and metastatic spread to the spine is one of its rare manifestations.^[12–15]^ Few reports of ACCs metastatic spread to the spine causing clinical symptoms have been documented so far. The location of the spinal lesion determines the neurological deficits, and there is a great deal of variability.^[16,17]^ Like most spinal disorders, spinal metastatic ACC may result in vertebral collapse, spinal instability, and progressive neurologic damage, which may cause local, radicular, or axial pain in addition to neurologic deficits from mild radicular weakness to paraparesis.^[18,19]^ Moreover, back pain is also a common symptom and it may make the differential diagnosis more difficult. As is consistent with our patient, spinal metastases are generally extradural and located most frequently at thoracic and lumbar spine.^[1–5,20–26]^

Clinical studies looking at metastatic ACC to the spine is lacking due to the extremely low incidence rate. Imaging studies including CT, MRI, and bone scan are nonspecific, making it difficult to differentiate metastatic spinal ACC from other common spinal lesions. However, imaging studies may play a crucial role in the decision making of surgical intervention. FDG-PET/CT scan is also insightful, as ACC usually exhibits high ^18^F-fluorodeoxyglucose uptake.^[1–3,18–20]^ Imaging should be followed by pathological confirmation, as the “criterion standard” diagnosis of ACC relies on pathological findings.^[1–5,27,28]^ Standard of care for advanced ACC (stage IV) is medical therapy in the form of mitotane.^[1–5,29]^ Mitotane is reported to be the only adrenal-specific and most effective therapeutic strategy currently available for patients with ACCs. Given its high recurrence rate, mitotane can be used either alone or in combination with cytotoxic drugs (doxorubicin, etoposide, cisplatin) from initial diagnosis.^[1–3,29,30–34]^ Research into newer therapies, including tyrosine kinase inhibitors (insulin-like growth factor 1 receptor) and rapamycin signaling components (mechanistic target of rapamycin) show some promise; however, it will still take several years from clinical application.^[1,5,31–34]^

Surgery is the best treatment of choice for metastatic spinal ACCs causing back pain, radiculopathy, and myelopathy. Surgical intervention in the case of advanced ACC with spinal metastases has met with some success, to date. Total resection of local regional lesions is associated with improved survival rates, according to several retrospective studies.^[3,32,33]^ Total or grossly total resection with stabilization is the most preferred intervention for lesions involving a single segment or two adjacent segments, as it is locally curative and provides good long-term outcomes.^[38]^ Debulking resection is also noted to be of benefit in cases whose medical treatment failed.^[3–5,32–34]^ However, surgical indications should be carefully considered for patients with spinal metastatic ACC, as the procedures are associated with prognosis of this rare entity.^[1–5,33–37]^

Osteoplasty by cement augmentation may be a treatment option for patients with metastatic ACC in the spine, who cannot undergo appropriate surgery or decline open surgery.^[33–38]^ However, we need to fully recognize the potential risk of complications in bone cement applications. The safety of this approach still needs to be confirmed in further studies with larger sample sizes and longer follow-up periods. One postoperative complication was cement leakage into the canal and subsequent spinal cord compression.^[16,17,37]^ Under this circumstance, surgical extent, cement volume, and postoperative complications are critical factors that need further investigation.^[37]^ The present case is the first reported so far that focused on patients with spinal metastasis from adrenocortical carcinoma which was treated by percutaneous vertebroplasty. It aimed to contribute to the appropriate surgical treatment of these patients.

The survival benefit of resection of spinal metastases is still unproven. However, such a procedure does have the benefit aiming at controlling residual tumor.^[38,39]^ The improved survival benefited from reducing the tumor burden, decompressing the spinal stenosis to alleviate radiculopathy or myelopathy, and facilitating subsequent adjuvant therapies. Radiotherapy has also shown promise as a treatment option for spinal metastatic ACC. Response rates to radiotherapy have been reported up to 42%.^[38,39]^ In fact, the German ACC team recommends that adjuvant radiotherapy be applied for lesions of stage III or of indeterminate resection margins following resection.^[39]^ In addition, PD-1, an immunoinhibitory receptor, has shown the ability to function as a marker for tumor prognosis.^[40,41]^ To date, the PD-1 and programmed death-ligand 1 (PD-L1) expression in ACCs and its prognostic significance are still uncertain. PD-1-positive or PD-L1-positive tumors may indicate immune active tumors that have a better response to anti-PD-1 and/or anti-PD-L1 therapies because they are correlated with the poor prognosis of many malignancies.^[40,41]^ As is reported in our case, anti-PD-1 therapy was performed postoperatively, and therapeutic effect has been achieved in clinical manifestations and imaging features.

## 4 Conclusion

Although uncommon, metastatic ACC of the spine should be part of the differential when the patient presents with neurological deficits or other symptoms. Clinical symptoms are generally the result of the tumor burden, and pathological results remain the “criterion standard” for diagnosing ACC. The treatment of spinal metastasis of ACC requires careful consideration of its aggressive nature. Complete resection, combined with radiotherapy or targeted PD-1/PD-L1 therapy, would be a logical approach to spinal metastasis of ACC. Osteoplasty by cement augmentation might also be good choices for treatment of spinal metastatic ACCs. With a multidisciplinary team approach, proper planning, and adequate perioperative medical management, metastatic ACC in the spine can be managed effectively.

## Acknowledgments

The authors thank their colleagues at the Department of Orthopaedic Surgery, Peking Union Medical College Hospital, Chinese Academy of Medical Sciences and Peking Union Medical College.

## Author contributions

**Conceptualization:** Shuzhong Liu, An Song, Yipeng Wang, Yong Liu.

**Funding acquisition:** Shuzhong Liu, Yipeng Wang, Yong Liu.

**Investigation:** Shuzhong Liu, Xi Zhou, An Song, Yong Liu.

**Resources:** Shuzhong Liu, Xi Zhou, Zhen Huo, Yong Liu.

**Supervision:** Yipeng Wang, Yong Liu.

**Writing – original draft:** Shuzhong Liu, Xi Zhou, An Song.

**Writing – review & editing:** Shuzhong Liu, Yipeng Wang, Yong Liu.

## References

[1] Singhal M Kang M Khadwal A An unusual presentation of congenital adrenocortical carcinoma: a case report and review of the literature. Cancer Imaging 2012;12:118–21. 22542918 10.1102/1470-7330.2012.0024 PMC3362873

[2] Ishida K Inoue Y Woodhams R Imaging findings of pelvic tumor thrombosis extending from sacral bone metastasis of adrenocortical carcinoma. Case Rep Radiol 2012;919603. 23326744 10.1155/2012/919603 PMC3541597

[3] Libe R Adrenocortical carcinoma (ACC): diagnosis, prognosis, and treatment. Front Cell Dev Biol 2015;3:45. 26191527 10.3389/fcell.2015.00045 PMC4490795

[4] Kebebew E Reiff E Duh QY Extent of disease at presentation and outcome for adrenocortical carcinoma: have we made progress? World J Surg 2006;30:872–8. 16680602 10.1007/s00268-005-0329-x

[5] Abiven G Coste J Groussin L Clinical and biological features in the prognosis of adrenocortical cancer: poor outcome of cortisol-secreting tumors in a series of 202 consecutive patients. J Clin Endocrinol Metab 2006;91:2650–5. 16670169 10.1210/jc.2005-2730

[6] Glover AR Ip JC Zhao JT Current management options for recurrent adrenocortical carcinoma. Onco Targets Ther 2013;6:635–43. 23776337 10.2147/OTT.S34956 PMC3681406

[7] Jarial KD Ahuja CK Mukherjee S Unusual cause of paraparesis in a patient with Cushing's syndrome. BMJ Case Rep 2016;2016:bcr2016217304. 10.1136/bcr-2016-217304 PMC5051364 27655879

[8] Janssen S Bartscht T Rades D Prognosis of patients with metastatic spinal cord compression from adrenocortical carcinoma. In Vivo 2016;30:717–9. 27566097

[9] Konstantinov AS Shelekhova KV Ectopic adrenal cortical adenoma in the spinal canal: a case report and a review of the literature. Arkh Patol 2016;78:44–8. 27296006 10.17116/patol201678344-48

[10] Maslin D Gounaris I Ng K Lesson of the month 2: cauda equina in Cushing's syndrome. Clin Med (Lond) 2016;16:88–90. 26833526 10.7861/clinmedicine.16-1-88 PMC4954344

[11] Lee D Yanamadala V Shankar GM Metastatic adrenal cortical carcinoma to T12 vertebrae. J Clin Neurosci 2016;27:166–9. 26765762 10.1016/j.jocn.2015.11.009

[12] Milgrom SA Goodman KA The role of radiation therapy in the management of adrenal carcinoma and adrenal metastases. J Surg Oncol 2012;106:647–50. 22488095 10.1002/jso.23096

[13] Schittenhelm J Ebner FH Harter P Symptomatic intraspinal oncocytic adrenocortical adenoma. Endocr Pathol 2009;20:73–7. 19039533 10.1007/s12022-008-9051-1

[14] Rodriguez FJ Scheithauer BW Erickson LA Ectopic low-grade adrenocortical carcinoma in the spinal region: immunohistochemical and molecular cytogenetic study of a pediatric case. Am J Surg Pathol 2009;33:142–8. 18941403 10.1097/PAS.0b013e318180deda PMC3427599

[15] Tauchmanovà L Pivonello R Di Somma C Bone demineralization and vertebral fractures in endogenous cortisol excess: role of disease etiology and gonadal status. J Clin Endocrinol Metab 2006;91:1779–84. 16522701 10.1210/jc.2005-0582

[16] Liu S Song A Zhou X Malignant pheochromocytoma with multiple vertebral metastases causing acute incomplete paralysis during pregnancy: Literature review with one case report. Medicine (Baltimore) 2017;96:e8535. 29095319 10.1097/MD.0000000000008535 PMC5682838

[17] Bartels RH van der Linden YM van der Graaf WT Spinal extradural metastasis: review of current treatment options. CA Cancer J Clin 2008;58:245–59. 18354080 10.3322/CA.2007.0016

[18] Else T Kim AC Sabolch A Adrenocortical carcinoma. Endocr Rev 2014;35:282–326. 24423978 10.1210/er.2013-1029 PMC3963263

[19] Allolio B Fassnach M Clinical review: adrenocortical carcinoma: clinical update. J Clin Endocrinol Metab 2006;91:2027–37. 16551738 10.1210/jc.2005-2639

[20] Solans R Vilardell M Beatriz Vázquez A Bone metastases as presentation of adrenocortical carcinoma. Med Clin (Barc) 2001;116:76–7. 11181278

[21] Marsden HB Jones PM Lees PD Late functioning adrenocortical carcinoma in a 5-year-old girl. Arch Dis Child 1978;53:341–2. 646449 10.1136/adc.53.4.341 PMC1544868

[22] Fassnacht M Allolio B Clinical management of adrenocortical carcinoma. Best Pract Res Clin Endocrinol Metab 2009;23:273–89. 19500769 10.1016/j.beem.2008.10.008

[23] Kuruba R Gallagher SF Current management of adrenal tumors. Curr Opin Oncol 2008;20:34–46. 18043254 10.1097/CCO.0b013e3282f301fd

[24] Fassnacht M Terzolo M Allolio B Combination chemotherapy in advanced adrenocortical carcinoma. N Engl J Med 2012;366:2189–97. 22551107 10.1056/NEJMoa1200966

[25] De Francia S Ardito A Daffara F Mitotane treatment for adrenocortical carcinoma: an overview. Minerva Endocrinol 2012;37:9–23. 22382612

[26] Aufforth RD Nilubol N Emerging therapy for adrenocortical carcinoma. Int J Endocr Oncol 2014;1:173–82. 25635221 10.2217/ije.14.13 PMC4307842

[27] Loblaw DA Perry J Chambers A Systematic review of the diagnosis and management of malignant extradural spinal cord compression: the Cancer Care Ontario Practice Guidelines Initiative's Neuro-Oncology Disease Site Group. J Clin Oncol 2005;23:2028–37. 15774794 10.1200/JCO.2005.00.067

[28] Crucitti F Bellantone R Ferrante A The Italian Registry for Adrenal Cortical Carcinoma: analysis of a multiinstitutional series of 129 patients. The ACC Italian Registry Study Group. Surgery 1996;119:161–70. 8571201 10.1016/s0039-6060(96)80164-4

[29] Habra M Ejaz S Feng L A retrospective cohort analysis of the efficacy of adjuvant radiotherapy after primary surgical resection in patients with adrenocortical carcinoma. J Clin Endocrinol Metab 2013;98:192–7. 23150683 10.1210/jc.2012-2367 PMC3537094

[30] Sabolch A Feng M Griffith K Adjuvant and definitive radiotherapy for adrenocortical carcinoma. Int J Radiat Oncol Biol Phys 2011;80:1477–84. 20675074 10.1016/j.ijrobp.2010.04.030

[31] Ofluoglu O Minimally invasive management of spinal metastases. Orthop Clin North Am 2009;40:155–68. 19064063 10.1016/j.ocl.2008.09.006

[32] Bròdano GB Cappuccio M Gasbarrini A Vertebroplasty in the treatment of vertebral metastases: clinical cases and review of the literature. Eur Rev Med Pharmacol Sci 2007;11:91–100. 17552138

[33] Anselmetti G Manca A Kanika K Temperature measurement during polymerization of bone cement in percutaneous vertebroplasty: an in vivo study in humans. Cardiovasc Intervent Radiol 2009;32:491–8. 19280257 10.1007/s00270-009-9509-7

[34] van der Linden YM Dijkstra SP Vonk EJ Prediction of survival in patients with metastases in the spinal column: results based on a randomized trial of radiotherapy. Cancer 2005;103:320–8. 15593360 10.1002/cncr.20756

[35] Gerszten PC Burton SA Ozhasoglu C Radiosurgery for spinal metastases: clinical experience in 500 cases from a single institution. Spine (Phila Pa 1976) 2007;32:193–9. 17224814 10.1097/01.brs.0000251863.76595.a2

[36] Patchell RA Tibbs PA Regine WF Direct decompressive surgical resection in the treatment of spinal cord compression caused by metastatic cancer: a randomised trial. Lancet 2005;366:643–8. 16112300 10.1016/S0140-6736(05)66954-1

[37] Liu S Zhou X Song A Successful treatment of malignant pheochromocytoma with sacrum metastases: A case report. Medicine (Baltimore) 2018;97:e12184. 30170467 10.1097/MD.0000000000012184 PMC6393136

[38] Klimo P Jr Thompson CJ Kestle JR A meta-analysis of surgery versus conventional radiotherapy for the treatment of metastatic spinal epidural disease. Neuro Oncol 2005;7:64–76. 15701283 10.1215/S1152851704000262 PMC1871618

[39] Wu J Wong R Johnston M Meta-analysis of dose-fractionation radiotherapy trials for the palliation of painful bone metastases. Int J Radiat Oncol Biol Phys 2003;55:594–605. 12573746 10.1016/s0360-3016(02)04147-0

[40] Wang Q Liu F Liu L Prognostic significance of PD-L1 in solid tumor: an updated meta-analysis. Medicine (Baltimore) 2017;96:e6369. 28471952 10.1097/MD.0000000000006369 PMC5419898

[41] Fay AP Signoretti S Callea M Programmed death ligand-1 expression in adrenocortical carcinoma: an exploratory biomarker study. J Immunother Cancer 2015;3:3. 25767716 10.1186/s40425-015-0047-3 PMC4357210

